# Research on the Magnetorheological Finishing Technology of a High-Steepness Optical Element Based on the Virtual-Axis and Spiral Scanning Path

**DOI:** 10.3390/mi15091154

**Published:** 2024-09-15

**Authors:** Chihao Chen, Chaoliang Guan, Meng Liu, Yifan Dai, Hao Hu

**Affiliations:** 1 College of Intelligent Science and Technology, National University of Defense Technology, Changsha 410073, China; chenchihao3218@nudt.edu.cn (C.C.); chlguan@nudt.edu.cn (C.G.); dyf@nudt.edu.cn (Y.D.); 2 National Key Laboratory of Equipment State Sensing and Smart Support, Changsha 410073, China

**Keywords:** MRF, high-steepness, virtual-axis, spiral scanning path, spherical/aspheric optical components

## Abstract

Magnetorheological finishing (MRF) of aspherical optical elements usually requires the coordination between the translational axes and the oscillating axes of the machine tool to realize the processing. For aspheric optical elements whose steepness exceeds the machining stroke of the equipment, there is still no better method to achieve high-precision and high-efficiency error convergence. To solve this problem, an MRF method combining virtual-axis technology and a spiral scanning path is proposed in this paper. Firstly, the distribution law of the magnetic induction intensity inside the polishing wheel is analyzed by simulation, the stability of the removal efficiency of the removal function within the ±7^∘^ angle of the normal angle of the polishing wheel is determined, and MRF is expanded from traditional single-point processing to circular arc segment processing. Secondly, the spiral scanning path is proposed for aspherical rotational symmetric optical elements, which can reduce the requirements of the number of machine tool axes and the dynamic performance of machine tools. Finally, an aspherical fused silica optical element with a curvature radius of 400 mm, K value of −1, and aperture of 100 mm is processed. The PV value of this optical element converges from 189.2 nm to 24.85 nm, and the RMS value converges from 24.85 nm to 5.74 nm. The experimental results show that the proposed combined process has the ability to modify curved optical elements and can be applied to ultra-precision machining of high-steepness optical elements.

## 1. Introduction

As advanced optical system technologies continue to achieve new milestones, the demand for diverse spherical and aspheric optical components, alongside their technical specifications, is concurrently escalating [1]. Aspherical optical elements have great advantages in improving the design freedom, imaging quality, and lightweight design of optical systems [2]. In the design of aviation and aerospace equipment and weapons, the window components are usually designed as high-steepness arched aspheric surfaces to meet the basic optical design requirements while also meeting the aerodynamic design requirements to achieve better flight control [3]. Due to the extremely stringent technical specifications of the scenarios in which they are used, these optics usually require complex and sophisticated processing and manufacturing processes to meet their accuracy requirements.

Magnetorheological finishing (MRF) is widely used in the polishing of various optical components due to its advantages of high machining accuracy, low subsurface damage, and high removal efficiency [4]. The conventional MRF uses the lowest normal point of the polishing wheel to achieve material removal of the workpiece [5]. Therefore, when processing aspherical optical components, it is usually necessary to cooperate between the translation axes (X/Y/Z) and the rotation axes (A/B) of the machine tool (five-axis linkage) and to achieve the removal of excess material on the surface in accordance with a certain path planning method. The high-steepness optical element generally refers to an optical element with an aspherical surface and large curvature change, and the swing angle of the A/B swing axis is generally required to exceed 25^∘^ in the process of MRF. Such optical components often exceed the motion stroke of the machine tool swing shaft during processing and generally need to be processed by disassembling the limiting device of the machine tool. This method does not guarantee the personal safety of the operator but also affects the stability of the surface rheological ribbon because of the large swing amplitude of the polishing wheel in the processing process, which, in turn, affects the actual accuracy of material removal.

To solve the technical problems of high-precision and high-efficiency convergence processing of high-steepness aspherical optical elements, many researchers have conducted in-depth studies. QED [6], as a global leader in MRF technology, pioneered virtual-axis technology, which extends the range of machining applicability of a machine tool by means of the polishing wheel’s own arc compensation technology without the need for additional mechanical axes and has integrated this technology into machines such as the Q-FLEX 300. Song et al. [7] investigated the magnetic induction intensity distribution of MRF polishing wheels and determined the angular intervals in which the removal function efficiency is stabilized by comparing the removal efficiency with the morphology of the removal function. Tao Zhang [8] proposed a method of combining virtual and mechanical axes, using the raster scanning path, realizing virtual axis machining of spherical optics and extending the travel of the machine tool’s mechanical axes. The results of the above research show that virtual axes are a proven technology for the machining of high-steepness aspherical optics and have been marketed to a certain extent.

Based on the above research results, this paper tries to combine virtual-axis technology with a spiral scanning path to realize MRF of aspherical optical elements with fewer machine axes. In conventional aspheric optics processing, raster scanning is usually used for path planning. This process requires the interplay between the machine’s translational axes (X/Y/Z) and the rotational axes (A/B). However, during the raster scanning process, the mass and inertia of the mechanical axes themselves [9] produce large acceleration and deceleration and mechanical vibration during the line change. These factors not only affect the positioning error between the polishing tool and the optics but also affect the stability of the polishing ribbon during the line change, which has an impact on the convergence of accuracy.

The combination of the spiral scanning path and virtual-axis technology proposed in this paper can effectively avoid the above-mentioned effects on polishing stability due to the acceleration and deceleration of the machine tool. For the machining of rotationally symmetric aspheric optical elements, the spiral scanning path requires only two translational axes (X/Z) and one rotational axis, the B-axis. Compared to raster scanning path planning, spiral scanning paths require fewer axes of machine motion, and vibration in the mechanical axes has less impact on machining. Moreover, the process only requires the polishing tool to move along the optics bus without changing lines, which requires less acceleration and deceleration of the machine. With the introduction of virtual-axis technology, only two translational axes (X/Z) are required to machine optical components whose steepness is within the stabilizing range of the removal function. For optics with a steepness greater than the stabilizing range of the removal function, the B-axis oscillation angle will be smaller than in conventional machining with the compensation of the virtual axes. This combined process not only realizes the machining of high steepness aspheric optical elements but also reduces the need for the number of mechanical axes in the machine tool, which is of great significance in enhancing the applicability of MRF.

## 2. Theoretical Analysis

### 2.1. Post-Processing

**Figure 1:**
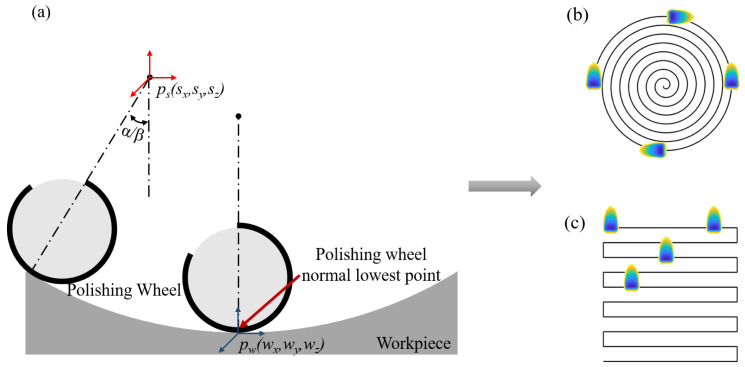
(**a**) Traditional machining. (**b**) Spiral scanning path. (**c**) Raster scanning path.

MRF usually requires the ribbon at the normal minimum point on the polishing wheel to contact each other with each discrete point on the surface of the workpiece at a certain depth of indentation to realize the process. This requires that the correct relative positional relationship between the polishing tool and the surface of the workpiece to be processed is always maintained to ensure that the relative clearance between the contact point of the polishing wheel and the workpiece does not change with the positional movement of the polishing tool [10]. Aspherical optics require the machine’s X, Y, Z, A, and B axes to work in conjunction with each other when machining using a raster scanning path. In the process of machining using the spiral scanning path, with the X, Z, and B axes of the machine tool cooperating with each other, the polishing tool only needs to do a simple curve movement along the workpiece bus to complete this kind of rotary symmetric workpiece processing. Spiral scanning path and raster scanning path are shown in Figure 1.

According to the basic principles of machine kinematics, it is assumed that in the initial state, the origin of the polishing wheel system coordinates coincide with the origin of the workpiece coordinates and that the polishing wheel traverses the surface of the optical element from the initial state in accordance with a certain path-planning method for all the discrete points of residence in the machining process. It is assumed that the position matrix of the stationary point in the work coordinate system is $p_{w}\left[w_{x},w_{y},w_{z}\right]$, and the vector matrix of the normal direction of the stationary point is $u_{w}\left[u_{x},u_{y},u_{z}\right]$. The position matrix of the machine’s translational X-, Y-, and Z-axis relative to the initial state during the machining of the polishing tool is $p_{s}\left[s_{x},s_{y},s_{z}\right]$. The angle matrix of the rotational A and B axes compared to the initial state is α and β (angles are determined positive or negative by the right-handed screw rule).

According to the principle of the multi-body model of the machine tool, the post-processing coordinate transformation matrix for the transformation from the coordinates of the workpiece to the coordinates of the machine tool motion is obtained after the motion calculation of the positional relationship between the polishing wheel and the workpiece:

(1) $$\begin{array}{c} \left\{\begin{array}{c} \alpha =\arcsin(u_{y})\\ \beta =\arctan(\frac{u_{x}}{u_{z}}) \end{array}\right.\\ \left\{\begin{array}{c} s_{x}=x_{w}+l_{b}\times \sin\beta \\ s_{y}=y_{w}-\left[l_{a}-l_{b}\times \left(1-\cos\beta \right)\right]\times \sin\alpha \\ s_{z}=z_{w}+\left[l_{a}-l_{b}\times \left(1-\cos\beta \right)\right]\times \cos\alpha -l_{a} \end{array}\right. \end{array}$$

In this formula, $l_{a}/l_{b}$ are the swing arm lengths of the A/B axes in relation to their rotational centers.

When the path planning method is raster scanning, the X, Y, Z, A, and B axes are required to coordinate and interact with each other in the processing of aspherical optical elements, and the post-processing is calculated and analyzed directly according to the above homogeneous transformation matrix. When spiral scanning is used for path planning, only the X, Z, and B axes need to work together. Since the motion coordination of the A and Y axes is not required, matrices α and $y_{w}$ in the calculation formula of post-processing currently are zero matrices, so the post-processing coordinate transformation matrix of the spiral scan path is as follows:

(2) $$\begin{array}{c} \left\{\beta =\arctan(\frac{u_{x}}{u_{z}})\right.\\ \left\{\begin{array}{c} s_{x}=x_{w}+l_{b}\times \sin\beta \\ s_{z}=z_{w}+l_{b}\times \left(\cos\beta -1\right) \end{array}\right. \end{array}$$

### 2.2. Virtual-Axis Technology

**Figure 2:**
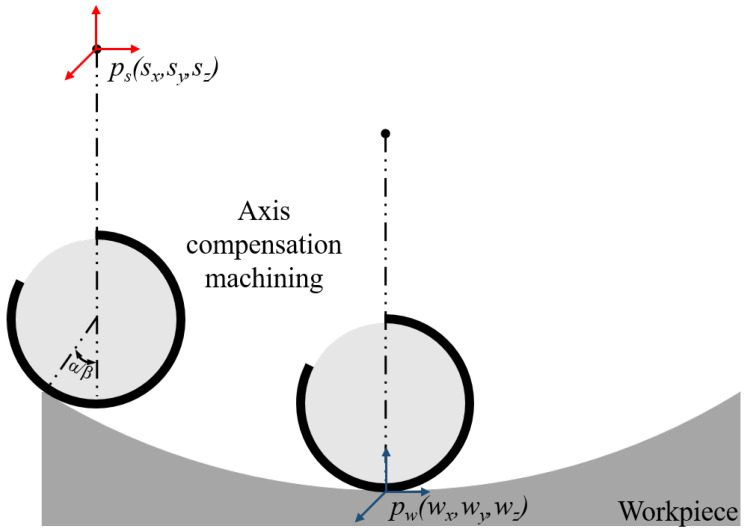
Virtual-axis technology.

Virtual-axis technology is based on machine tool multi-axis linkage machining regarding the geometric symmetry of the polishing wheel arc and the symmetry distribution law of the gradient magnetic induction intensity [11]. In the polishing process, the lowest point of the polishing wheel no longer uses normal contact machining, instead using the ribbon in a certain range of normal vectors and the workpiece surface shape of the normal vector of the fit between the match, as shown in Figure 2.

At this point, the processing range of the polishing wheel is extended from a point to a section of arc, like the “arc interpolation” technology used in turning [12], which can realize the processing of complex surfaces with fewer axes of motion. To realize the compensating effect of the virtual axis on the swing of the rotational axis, the swing compensation of the rotation B-axis is realized by the interplay between the translational movement of the X/Z axes and the expanding arc section. In actual machining, the rotational B-axis no longer needs to be deflected around its rotational center, but it still needs to compensate for the X/Z axis movement of this angle matrix β. Without considering machine tool error, the coordinate transformation matrix of each axis of the machine tool during the motion can be found as follows:

(3) $$\left\{\begin{array}{c} \beta =\arctan(u_{x}/u_{z})\\ s_{x}=x_{w}-R\cdot \sin\beta \\ s_{z}=z_{w}+R\cdot \left(\cos\beta -1\right) \end{array}\right.$$

In this formula, *R* is the radius of the polishing wheel. With virtual-axis technology, optical elements whose steepness does not exceed the travel of the machine’s oscillating axis can be machined by omitting the oscillating fit of the B-axis and by simply compensating for the movement of the polishing wheel in the X/Z-axis and the expansion of the arc. For optical components whose steepness exceeds the machine’s swing travel, the rotary axis only needs to swing to a specific safety angle during machining, and the remaining swing travel is compensated for by the virtual axis.

## 3. Magnetic Induction Intensity Distribution Simulation

MRF is realized on the principle that the magnetorheological fluid solidifies rapidly due to the rheological effect under the action of a gradient magnetic induction intensity [13], forming a flexible polishing ribbon at the bottom of the polishing wheel. The ribbons and the workpiece contact each other at a certain depth of indentation, and material removal from the surface of the optical element is realized under the action of shear force. In the field of optical machining, the basic principle of trimming for MRF is Preston’s assumption [14] that the actual amount of material removed is determined by the following equation:

(4) Δh=kpv

In this equation, Δh is the theoretical material removal, *k* is Preston’s constant, *p* is the positive pressure between the optical part and the polishing tool, and *v* is the relative speed of motion between the optical part and the polishing tool.

The positive pressure *p* between the optical element and the polishing tool in MRF is determined by the following equation [15]:

(5) $$p=p_{l}+p_{m}$$

In this formula, $p_{l}$ is the liquid flow pressure, and $p_{m}$ is the magnetizing pressure. For MRF, the flow pressure $p_{l}$ [16] of the magnetorheological fluid at each position of the polishing wheel is essentially constant due to the geometrical symmetry of the wheel itself and the stability of the rotational speed during the polishing process. Thus, the main factor affecting the positive pressure *p* is the distribution of the magnetization pressure $p_{m}$ generated by the magnetorheological fluid under the gradient magnetic induction intensity of the expanding arc portion used in the virtual-axis technology. The variation of the positive pressure *p* during machining is manifested as a change in the efficiency of the removal function caused by a change in the distribution of the magnetic induction intensity in the arc portion of the segment.

**Figure 3:**
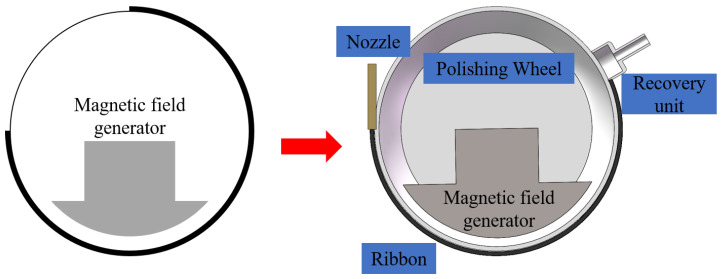
Electromagnet generator body part.

Based on the above analysis, the magnetic induction intensity simulation of the main part of the electromagnet generator used was carried out using COMSOL5.6 software to analyze the distribution of its magnetic induction intensity. A simplified model of the main part of the electromagnet generator is shown in Figure 3 below.

The relevant parameters in the simulation are shown in Table 1 below.

**Figure 4:**
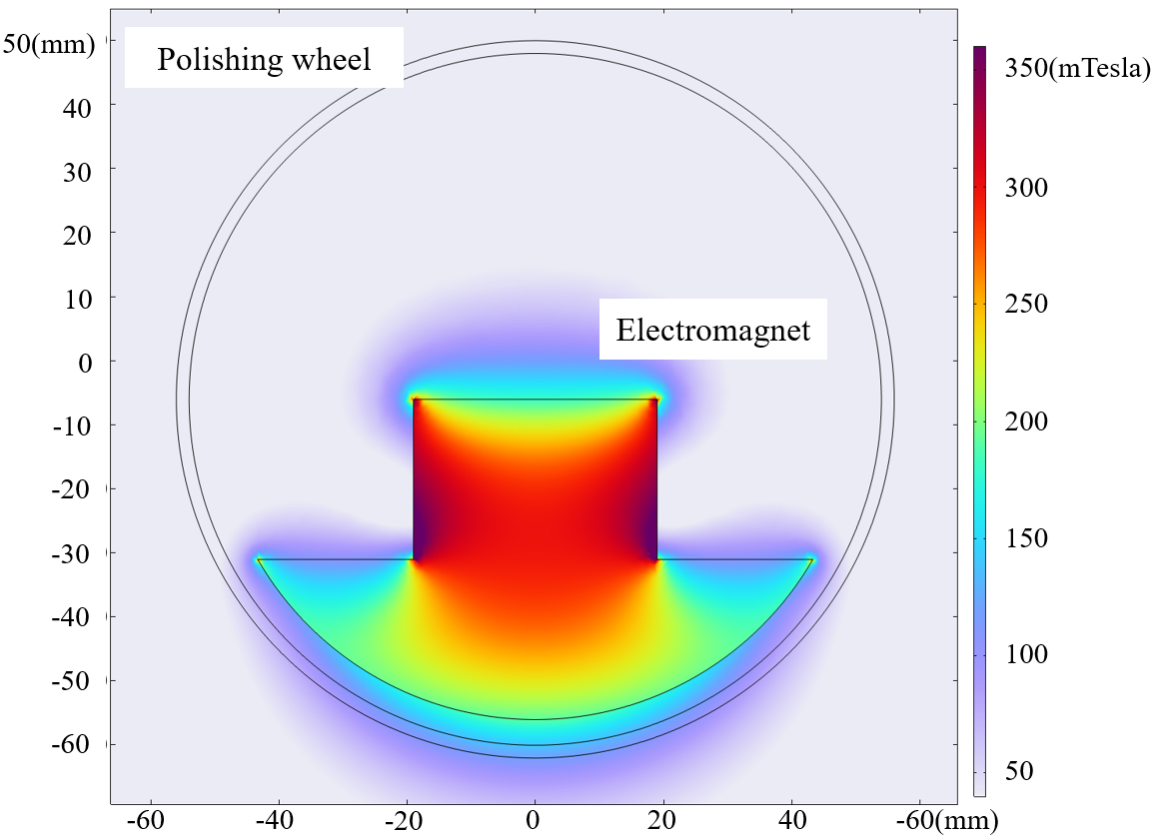
Magnetic induction intensity distribution.

In the actual structure, the electromagnet is placed inside the polishing wheel after being energized to generate a gradient magnetic induction intensity within a certain space. At this time, the magnetorheological fluid is sprayed out through the nozzle under the action of the magnetic induction intensity rapidly occurring MRF and attached to the surface of the wheel. This adds to the polishing process on the other side of the recycling device recovered by the recycling system, e.g., the main analysis of the distribution of the magnetic induction intensity on the outside of the polishing wheel. The simulation results are shown in Figure 4.

**Figure 5:**
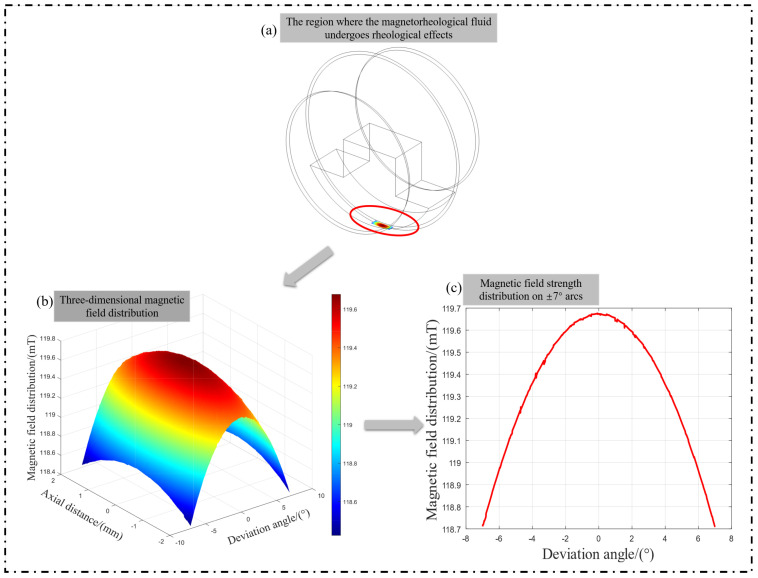
Magnetic induction intensity change curve.

Taking the magnitude of the magnetic induction intensity at the lowest point as a reference, the variation curve of the magnetic induction intensity within ±7^∘^ is shown in Figure 5. Figure 5a describes the spatial assembly relationship between the electromagnet and the polishing wheel in the internal structure of the magnetorheological polishing wheel. Figure 5b mainly shows the simulation of 3D magnetic induction intensity distribution in the space range of 2 mm width at the bottom of the polishing wheel and arc angle ±7^∘^ (in actual processing, the width of the magnetorheological ribbon is roughly 2 mm). Figure 5c mainly illustrates the parameter variations in the overall machining area in terms of the distribution of the magnetic induction intensity on the most central line within a certain angle to the left and right of symmetry normal to the lowest point of the polishing wheel.

The above simulation results show that a 100 mm polishing wheel in the existing magnetic induction intensity generator, ±7^∘^ within the magnetic induction intensity variation error of 0.7% can meet the use of demand. Through the post-processing analysis and calculation of the machine tool and the simulation analysis of the magnetic induction intensity of the polishing wheel, the theoretical feasibility of virtual-axis technology can be determined.

### Modification Ability Analysis of the Magnetorheological Removal Function

**Figure 6:**
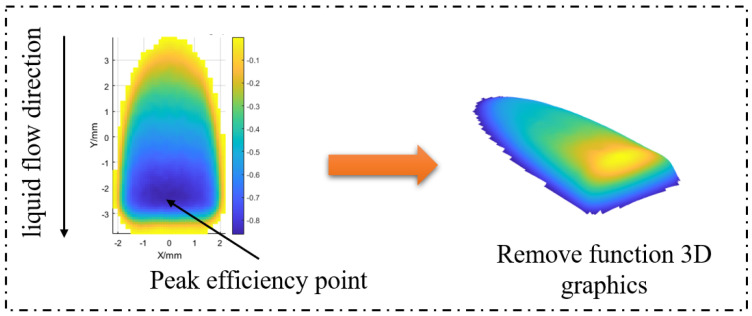
Inverted D shape.

The geometric shape, removal efficiency, surface roughness after polishing, cut-off frequency, and other parameters of the removal function are usually introduced in the MRF process so as to better quantify the modification ability of a removal function with different shapes and specifications. The geometry mainly evaluates the length and width of the removal function, as well as its unique inverted D shape appearance(as shown in the Figure 6). Removal efficiency mainly includes peak removal efficiency and volume removal efficiency, of which the peak removal efficiency refers to the removal function of the effective material and the removal range of the highest efficiency, where the unit is μm/min. For the volume removal efficiency for the removal function in the unit time of the total volume of material removed, the unit is mm^3^/min.

The above parameters can visualize the relationship between the size of the removal function and the removal efficiency. According to the existing experience, the smaller the size of the removal function, the lower the removal efficiency. However, in high-precision machining, the face-shape convergence ability of the small-size removal function tends to outperform that of the large-size removal function. To reveal the underlying principle of this phenomenon, Hu [17] proposed to define the modification ability of the removal function as the frequency corresponding to the sinusoidal surface error so that the removal function can completely correct. The basic principle is to calculate the one-dimensional or two-dimensional Fourier amplitude spectrum normalization of the removal function [18]. Taking the Gaussian removal function as an example, the expression of the one-dimensional removal function in the X direction is as follows:

(6) $$r\left(x\right)=\frac{B}{\sqrt{2\pi }\sigma }\times e^{-\frac{x^{2}}{2\sigma^{2}}}$$

After normalizing the one-dimensional Fourier amplitude spectrum of the above equation, the frequency corresponding to a certain percentage of the amplitude spectrum falling to the peak is chosen as the cut-off frequency of the removal function, which is usually considered to be able to effectively correct the surface shape error below this cut-off frequency.

## 4. Experimental Verification

### 4.1. Removal Function Stability Verification

According to the previous magnetic induction intensity simulation results, the removal function of a flat fused silica glass was extracted within the range of ±7^∘^ of the polishing wheel, and the material removal efficiency of each removal function was calculated. The removal function parameters used are as follows in the Table 2.

**Figure 7:**
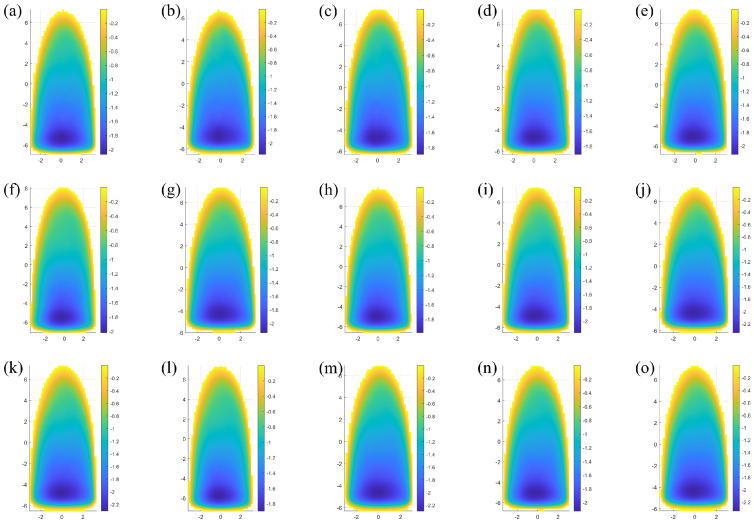
Geometric shape. (**a**) Removal function of 0^∘^ angle, (**b**–**h**) removal function of 1^∘^∼7^∘^ angle, and (**i**–**o**) −1^∘^∼−7^∘^.

According to the above experimental parameters, a total of 15 removal functions were extracted, and the surface shapes of each removal function are shown in Figure 7.

Firstly, the removal efficiency of the removal function under each swing angle is calculated, the removal efficiency of the removal function under 0^∘^ swing angle is taken as the benchmark, the deviation size of the removal efficiency of the removal function under each swing angle is calculated, and the removal efficiency and deviation of each removal function are shown in Table 3.

**Figure 8:**
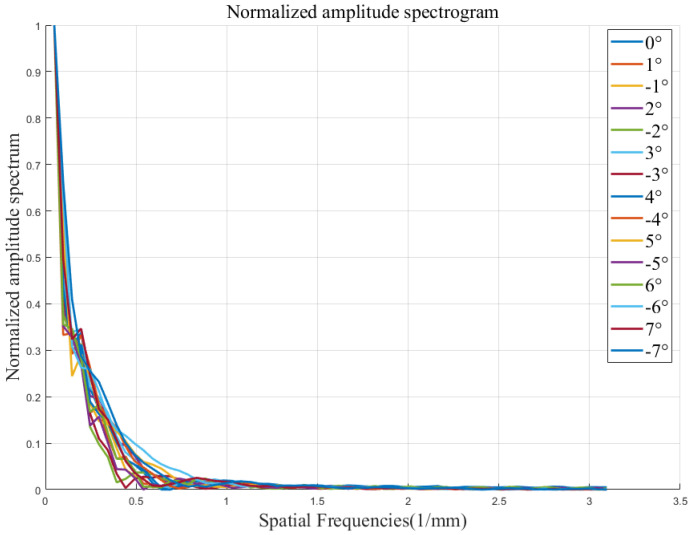
One dimensional Fourier amplitude spectrum normalization curve.

The one-dimensional Fourier amplitude spectrum normalization calculation was carried out for the above 15 removal functions, and the amplitude spectra are shown in the Figure 8.

By comparing the removal efficiency, geometry, and one-dimensional Fourier amplitude spectrum of the above 15 removal functions, the following conclusions can be drawn: in the angular range of ±7^∘^, the maximum value of the removal function efficiency change error is 1.68%, which is still within the acceptable error range, the removal function one-dimensional Fourier amplitude spectrum has the same trend of change, and it has the ability to correct more than 2 mm frequency band error. Therefore, in the actual processing, the arc segment within the angle range of ±7^∘^ of the polishing wheel can be used for polishing processing.

### 4.2. Virtual-Axis Machining Validation

**Figure 9:**
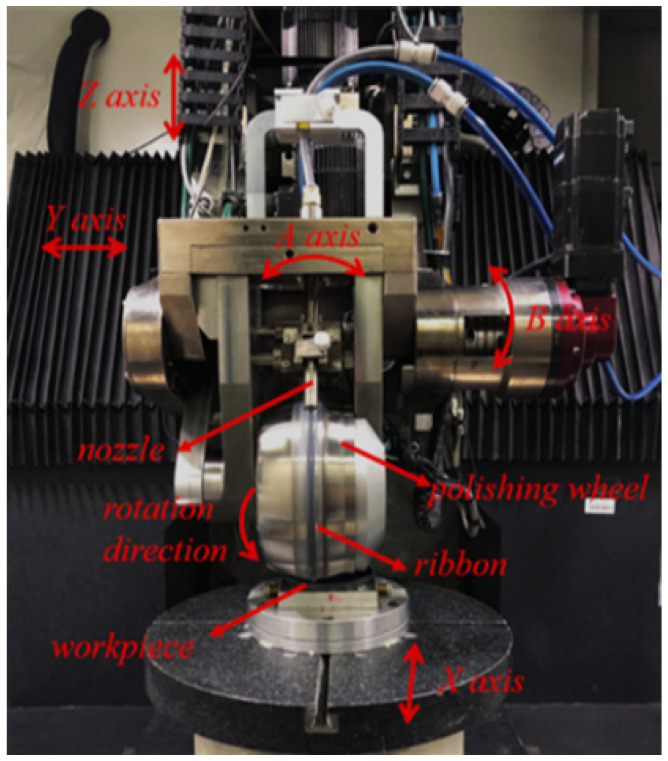
Self-developed MRF machine tool (KDUPF650-7).

To verify the correctness of the theory of virtual axis machining, a piece of a convex spherical surface with a radius of curvature of 400 mm, K value −1, and caliber of 100 mm is prepared for machining verification using ultra-precision grinding, stress disk polishing, and other processes. The equipment used is the KDUPF-650 [19], which was developed by our faculty team. The axes and related structures are shown in Figure 9.

**Figure 10:**
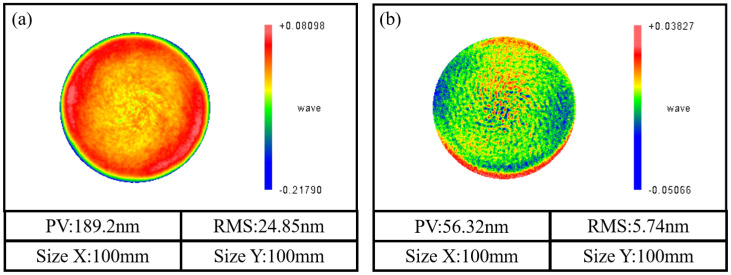
Surface error distribution before and after machining: (**a**) before processing and (**b**) after processing.

The process adopts 0.25 mm pressure depth and discrete processing according to the 0.5 mm spacing of the face shape, selecting the removal function with a swing angle of 0^∘^ for the residence time solution. Using a combination of helical scanning path planning and virtual-axis technology, the overall PV converges from 189.2 nm to 24.85 nm, and the RMS converges from 24.85 nm to 5.74 nm after 2 h of processing iterations. The machining results are shown in Figure 10.

**Figure 11:**
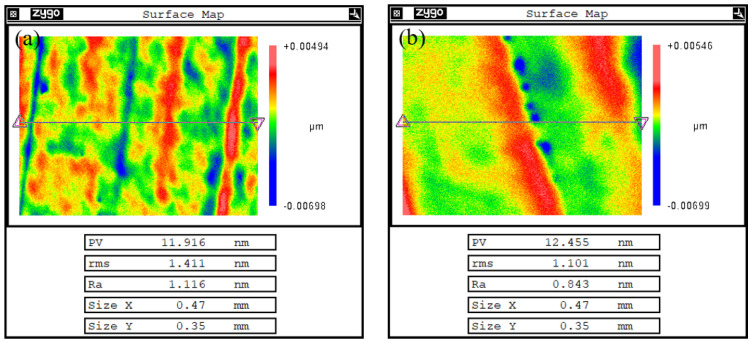
Roughness before and after machining: (**a**) before processing and (**b**) after processing.

In optical processing, the base roughness of optical elements is usually ensured by the pre-polishing process of MRF. Although optical elements already need to have high precision before entering MRF, MRF itself still has a certain enhancement effect on the surface roughness of the optical elements, so this paper includes the changes in the surface roughness of the optical elements before and after polishing in the discussion in order to further verify the feasibility of the process. Figure 11 shows the roughness measurement results before and after MRF polishing using a Zygo white light interferometer (Manufactured by Zygo, Middlefield, CT, USA) with a 20× lens. The measurement results show that the surface roughness of the optical element after MRF increased from the original Ra1.116 nm to 0.843 nm.

## 5. Discussion and Conclusions

In this paper, an MRF process route combining a virtual-axis and spiral scanning path is proposed, which can be applied to all kinds of curved optical elements, especially the high-precision machining of the surface of high-steepness optical elements. The main contributions and achievements of this paper are as follows.

Firstly, the distribution error of the magnetic induction intensity of the polishing wheel is less than 0.7% in the angle range of ±7^∘^ through modeling and simulation analysis. This result meets the requirement of the rheological effect of magnetorheological fluid theoretically. According to the simulation results, the removal functions at different radian positions of the polishing wheel were extracted, and the corresponding removal efficiency, normalized Fourier spectrum, and other evaluation indexes were calculated. The results in Figure 6 and Table 2 show that the geometric shapes of the removal functions at different angle positions of the polishing wheel maintain good consistency (the shapes are slender and inverted D shape), and the change in the efficiency of the removal function (the maximum value is 1.68%) is within the acceptable error range. The Fourier spectrum diagram in Figure 8 shows that the removal function of the polishing wheel at different angular positions has the ability to correct the space sine error of more than 2 mm. The above results determine the stability of the removal efficiency of the removal function on the arc section of the polishing wheel with ±7^∘^.

Secondly, based on the basic principle of virtual-axis technology, the post-processing coordinate transformation matrix corresponding to the spiral scanning path is reconstructed and calculated. The results of Figure 9 and Figure 10 verify the correctness of the post-processing transformation matrix of spiral scanning path planning based on virtual-axis technology, which can ensure the stable pose relationship between the polishing wheel and the workpiece during the machining process.

Finally, the effectiveness of the proposed virtual axis and spiral scanning path combination process was verified through processing experiments. By combining spiral scanning path planning with virtual-axis technology, the overall surface form PV converged from 189.2 nm to 56.32 nm and RMS converged from 24.85 nm to 5.74 nm after 2 h of processing iterations.At the same time, the roughness of the workpiece surface was increased from 1.116 nm to 0.843 nm.

The process route proposed in this paper effectively reduces the number of motion axes required for machine tools and minimizes dynamic performance demands during processing while simultaneously broadening the range of typical components that can be processed by existing equipment, thereby offering a novel approach to magnetorheological processing of high-steepness optical elements.

## Figures and Tables

**Table 1:** Electromagnet simulation parameters.

Polishing Wheel Size	Magnetic Induction Intensity	Magnetic Medium
100 mm	140 mTesla	Air

**Table 2:** Electromagnet simulation parameters.

Polishing Wheel Radius	Polishing Wheel Ratation Speed	Liquid Viscosity	Liquid Flow Rate	Ribbon Depth	Solenoid Current
100 mm	180 rpm	180 Pa·s	90 L/min	0.2 mm	6.5 A

**Table 3:** Efficiency and deviation of each removal function.

Swing Angle/^∘^	Volume Removal Efficiency/(10^7^ ∗ μm^3^/min)	Removal Efficiency Bias
0	7.13	/
1	7.02	1.54%
2	7.08	0.7%
3	7.02	1.54%
4	7.01	1.68%
5	7.09	0.56%
6	7.02	1.54%
7	7.06	0.98%
−1	7.06	0.98%
−2	7.10	0.42%
−3	7.04	1.26%
−4	7.06	0.98%
−5	7.12	0.14%
−6	7.04	1.26%
−7	7.07	0.84%

## Data Availability

The data presented in this study are available upon request from the corresponding author. The data are not publicly available because they are part of an ongoing study.
